# Supplementary material for: Randomized, observer-blind, controlled Phase 1 study of the safety and immunogenicity of the Na-GST-1/Alhydrogel hookworm vaccine with or without a CpG ODN adjuvant in hookworm-naïve adults
Source: PLoS Negl Trop Dis. 2024 Dec 30;18(12):e0012788. doi: 10.1371/journal.pntd.0012788 (PMC11717351; doi:10.1371/journal.pntd.0012788)
Supplement: S1 Table — (DOCX) [file pntd.0012788.s001.docx]

**S1 Table. Number and percentage of participants experiencing unsolicited related* adverse events by maximum severity and vaccine group.**

| **Severity** | **30µg *Na-*GST-1/Alhydrogel + 500µg CpG 10104**  (N=8) | | **100µg *Na-*GST-1/Alhydrogel**  (N=8) | | **100µg *Na-*GST-1/Alhydrogel + 500µg CpG 10104**  (N=8) | | **Overall**  (N=24) | |
| --- | --- | --- | --- | --- | --- | --- | --- | --- |
|  | **Events** | **n (%)** | **Events** | **n (%)** | **Events** | **n (%)** | **Events** | **n (%)** |
| Mild | 7 | 5 (62.5%) | 14 | 6 (75.0%) | 12 | 5 (62.5%) | 33 | 16 (66.6%) |
| Moderate | 2 | 1 (12.5%) | 0 | 0 (0.0%) | 0 | 0 (0.0%) | 2 | 1 (4.2%) |
| Severe | 0 | 0 (0.0%) | 0 | 0 (0.0%) | 0 | 0 (0.0%) | 0 | 0 (0.0%) |
| *Note: N = Number of participants in the Safety Population who received the specified vaccine. The number (n) and percentage (%) are for participant level. A participant was counted only once per severity grade. For the number of events, a participant could be counted multiple times.*  **Definitely, probably, or possibly related to study vaccine.* | | | | | | | | |
