# Supplementary material for: Randomized, observer-blind, controlled Phase 1 study of the safety and immunogenicity of the Na-GST-1/Alhydrogel hookworm vaccine with or without a CpG ODN adjuvant in hookworm-naïve adults
Source: PLoS Negl Trop Dis. 2024 Dec 30;18(12):e0012788. doi: 10.1371/journal.pntd.0012788 (PMC11717351; doi:10.1371/journal.pntd.0012788)
Supplement: S2 Table — (DOCX) [file pntd.0012788.s002.docx]

**S2 Table. Clinical laboratory adverse events by vaccine group after any dose.**

| **Parameter** | **30µg *Na*-GST-1/ Alhydrogel + 500µg CpG 10104**  (N=8) | | **100µg *Na*-GST-1/ Alhydrogel**  (N=8) | | **100µg *Na*-GST-1/ Alhydrogel + 500µg CpG 10104**  (N=8) | | **Overall**  (N=24) | |
| --- | --- | --- | --- | --- | --- | --- | --- | --- |
|  | **Events** | **n (%)** | **Events** | **n (%)** | **Events** | **n (%)** | **Events** | **n (%)** |
| Any Clinical Laboratory AE | 4 | 2 (25.0%) | 5 | 4 (50.0%) | 11 | 3 (37.5%) | 20 | 9 (37.5%) |
| Decreased WBC Count | 2 | 1 (12.5%) | 1 | 1 (12.5%) | 4 | 2 (25.0%) | 7 | 4 (16.7%) |
| Decreased ANC | 0 | - | 0 | - | 4 | 2 (25.0%) | 4 | 2 (8.3%) |
| Decreased Hemoglobin | 1 | 1 (12.5%) | 2 | 2 (25.0%) | 1 | 1 (12.5%) | 4 | 4 (16.7%) |
| Decrease in Hemoglobin from Day 0 | 0 | - | 0 | - | 1 | 1 (12.5%) | 1 | 1 (4.2%) |
| Increased ALT | 1 | 1 (12.5%) | 0 | - | 1 | 1 (12.5%) | 2 | 2 (8.3%) |
| Increased ANA Titer | 0 | - | 2 | 2 (25.0%) | 0 | - | 2 | 2 (8.3%) |
| *Note: N = Number of participants who received the specified vaccine. The number (n) and percentage (%) are for participant level. For the number of events, a participant could be counted multiple times.* | | | | | | | | |
