# Supplementary material for: Randomized, observer-blind, controlled Phase 1 study of the safety and immunogenicity of the Na-GST-1/Alhydrogel hookworm vaccine with or without a CpG ODN adjuvant in hookworm-naïve adults
Source: PLoS Negl Trop Dis. 2024 Dec 30;18(12):e0012788. doi: 10.1371/journal.pntd.0012788 (PMC11717351; doi:10.1371/journal.pntd.0012788)
Supplement: Attachment — Submitted filename: Response to Reviewers.docx [file pntd.0012788.s003.docx]

**Response to Reviewers**

Manuscript Number: **PNTD-D-24-01471**

Manuscript Title: **“Randomized, observer-blind, controlled Phase 1 study of the safety and immunogenicity of the *Na*-GST-1/Alhydrogel hookworm Vaccine with or without a CpG ODN adjuvant in hookworm-naïve adults”**

**Reviewer #1:**

1. **I would ask the authors to give more background on CpG 10104 (this is effectively a 10104 study). Is this first in human? If so, why no adjuvant alone group? If not, please describe and reference previous work.**

*Authors’ response*: Thank you for the thorough review of our manuscript. Yes, this study was the first time that the combination of *Na-*GST-1/Alhydrogel was tested in humans, although *Na*-GST-1/Alhydrogel had been tested in several Phase 1 trials before this, either alone or in combination with an aqueous formulation of glucopyranosyl lipid A. This fact has been stated in the first line of the Discussion. Given that adjuvants alone (i.e., without any antigen) would not be expected to induce antigen-specific immune responses, the utility of including an antigen-alone group in the study is unclear. Given that *Na-*GST-1/Alhydrogel had already been tested in humans (as referenced in the second paragraph of the Introduction [1-3]), and the aim of the study reported herein was to potentially improve the immunogenicity of the vaccine without compromising its safety or reactogenicity profiles, we felt that *Na-*GST-1/Alhydrogel itself would be a more appropriate comparator than placebo or adjuvant alone.

2. **What animal studies have been done with 10104?**

*Authors’ response*: Preclinical immunogenicity and toxicology animal studies of recombinant *Na*-GST-1 adjuvanted with Alhydrogel with or without the addition of CpG 10104 have been conducted in BALB/C mice and Sprague-Dawley rats, respectively. Detailed descriptions of these studies were included in the Investigational New Drug (IND) application to the US Food and Drug Administration for this vaccine and were deemed by this agency as being sufficient to permit testing in humans. A brief description of the toxicology study results has been added to the Methods section (Lines 120-123), as described in the response to the following comment of the Reviewer.

3. **How about toxicology?**

*Authors’ response*: Prior to initiating the study reported herein, a toxicology study was performed in Sprague-Dawley rats according to current Good Laboratory Practice, as required by US regulations governing Investigational New Drug applications. This study did not indicate any safety issues with *Na*-GST-1 adjuvanted with Alhydrogel with or without the addition of CpG 10104. There were no observed adverse effects on survival, body weights, or organ weights or any clinical signs of toxicity or abnormal gross necropsy findings. A brief statement regarding this has been added to the Methods section (Lines 120-123).

4. **Although looks safe, it is of note that with only 16 people getting 10104, one had tenderness at injection site that lasted for a month, and another person was classified as a “severe” local reaction. Would suggest stating that in discussion and say this will need to be followed in future studies.**

*Authors’ response*: The reviewer is correct in noting that one of 16 recipients of *Na*-GST-1/Alhydrogel plus CpG 10104 had injection site tenderness that the participant reported for 31 days; however, this reaction only occurred after the first of the three injections and was never more than mild in intensity. This same participant did not report any tenderness after the second injection and had mild tenderness of 3 days’ duration after the third injection. Additionally, the severe local reaction in another participant consisted of severe injection site tenderness in a participant after the second injection that did not recur after the third injection, and that lasted only 2 days. Nevertheless, a statement has been added to the first paragraph of the Discussion (Lines 380-382), as suggested by the reviewer, acknowledging that the sample size in this study was small and that surveillance for safety must continue in future studies.

5. **The authors give references for other CpGs, but the pertinent issue here is 10104, so please give more details.**

*Authors’ response*: The clinical trial that we are reporting in this manuscript was the first time that CpG 10104 was tested in humans. As such, references could not be included as requested.

6. **Please discuss what % Hw Na vs Ad.**

*Authors’ response*: *Necator americanus* is the most prevalent and widespread hookworm species infecting humans, accounting for an estimated 80% of infections [4]. A statement to this effect has been added to the second paragraph of the Introduction.

7. **Is GST-1 Conserved? Would an anti-GST protect against both, or are we only going after 1 of the 2 main global causes even if all works well.**

*Authors’ response*: *Na*-GST-1 homologues have been cloned from *A. ceylanicum* (*Ay*-GST-1) and *A. caninum* (*Ac*-GST-1). Alignment of the three available hookworm GST-1’s reveals 70-83% sequence identity and 87-92% similarity. Like recombinant *Na*-GST-1, recombinant *Ac*-GST-1 exhibits high affinity to heme, the toxic byproduct of hemoglobin digestion. Vaccination of dogs with recombinant *Ac*-GST-1 results in significant reduction in mean worm burden and egg counts compared to controls; hamsters vaccinated with *Ac*-GST-1 exhibited 53.7% cross-protection against heterologous *N. americanus* larvae challenge [5, 6]. Finally, anti-*Ac*-GST-1 rabbit serum recognizes native *Ay*-GST-1 and *Na*-GST-1 in the extracts of adult hookworms, indicating the cross-reactivity of antibodies recognizing *Ac*-GST-1 and *Ay*-GST-1/*Na*-GST-1.

Although the GST homologue from *A. duodenale* has not yet been cloned, based on sequence data, the percentage of identity between *Na*-GST-1 and its *A. duodenale* homologue ranges from 55% to over 76% depending on the isolate [7].

Due to high sequence homology of GSTs among *Necator* and *Ancylostoma* *spp*. and cross-reaction and cross-protection between *Na*-GST-1 and *Ac*-GST-1, it is reasonable to believe that immunization with *Na*-GST-1 might not only protect humans against *N. americanus* infection, but possibly against other hookworm infections including *A. duodenale*. However, this hypothesis needs to be confirmed by cloning and testing the GST-1 homologue (*Ad*-GST-1) from *A. duodenale* and assessing for efficacy in field clinical trials. A paragraph describing this hypothesis has been added to the Discussion (Lines 460-465).

8. **Please help reader with anti-GST background: has animal variants shown protection in their appropriate animal models (example dog or cat or other); if that work not done, would state.**

*Authors’ response*: Preclinical animal testing of recombinant GST-1 has been performed in dogs and Syrian Golden hamsters. These showed a significantly lower fecal egg counts in vaccinated animals after challenge infection compared to placebo or adjuvant alone. These results have been previously reported and referenced in published manuscripts of prior Phase 1 trials of *Na*-GST-1/Alhydrogel that have been referenced in the current paper [1-3], so it was not felt necessary to recapitulate these findings herein.

9. **Although CpG helps, as expected there is a decline in IgG that would kinetically map (even after after 3 doses with two adjuvants and high dose vaccine) to being back to baseline in 18-24 months; would at least point that out in discussion since even if can get by with 2 initial doses, this strategy may be looking at yearly or q24 month boosting to maintain meaningful Ab levels; this would be difficult with kids in LMIC setting since not EPI, although could be combined with annual MDAs; would discuss.**

*Authors’ response*: The reviewer is correct in observing the decline in antigen-specific IgG after the administered vaccinations until the end of the observation period in this study. It should be noted that this study was performed in a non-endemic setting and in adults, and therefore may not be representative of vaccine responses or kinetics in younger individuals living in endemic areas, who are the eventual target population for a hookworm vaccine. In addition to differences in age, repeated exposure to the parasite in endemic areas may result in “natural boosting” of the IgG response; in fact, this has been observed in the controlled human infection model of hookworm that is being used to assess the efficacy of candidate hookworm vaccines such as *Na*-GST-1/Alhydrogel plus CpG 10104. However, these results indicating boosting of anti-*Na*-GST-1 IgG responses by an infectious challenge have not yet been published and therefore cannot be referenced in this manuscript. Nevertheless, a hypothetical statement regarding the potential for boosting from natural exposure has been added to the Discussion (Lines 452-459).

10. **Please give CTN for the endemic zone study that the paper references twice and says has been initiated.**

*Authors’ response*: The ClinicalTrials.gov number for the Phase 1 trial of *Na*-GST-1/Alhydrogel with or without CpG 10014 in Gabonese adults has been added to concluding paragraph of the manuscript, as suggested.

11. **Can the authors more clearly state how many Hw vaccines are in development and their stage? Formulations and different teams are fluid, and would help reader.**

*Authors’ response*: Currently, only 2 hookworm vaccine antigens (*Na*-GST-1 and *Na*-APR-1(M74) are in clinical development. This is stated in the second paragraph of the Introduction.

12. **Overall, important work that is encouraging that 10104 assists.**

*Authors’ response*: Thank you, we agree!

**Reviewer #2:** **The study by Diemert et al, present safety results from a vaccination with GST-1 as an anti-hookworm vaccine. The novelty reside in the investigation of CpG as a novel adjuvant to boost antibody production. Interestingly, the authors show that Alum +CpG do boost antibody production and is well tolerated. A question of interest to answer in the future would be whether this increase in antibody is also reflected in an increase of neutralizing antibody. The authors could consider an *in vitro* assays with *Necator americanus* or *Nippostrongylus brasiliensis* as a surrogate and study the parasite viability in presence of blood and increasing concentration of serum isolated from vaccinated patients.**

*Authors’ response*: Thank you for this favorable review of the manuscript. Indeed, it would be interesting to assess the functionality of the antibodies induced by the *Na*-GST-1/Alhydrogel + CpG vaccine formulation using an in vitro neutralization assay. Our group has attempted to develop an in vitro assay to investigate if anti-*Na*-GST-1 IgG antibodies induced by vaccination are able to inhibit the activity of native *Na*-GST-1 enzyme activity; however, to date, this type of assay has proven difficult to standardize. Incubation of vaccine-induced anti-*Na*-GST-1 IgG antibodies with live adult *N. americanus* worms *in vitro* would not be expected to yield any meaningful results, given that the antibodies are hypothesized to inhibit the blood feeding pathway of the adult worms, which would only occur in a parasitized host intestinal tract. For this reason, we have developed a controlled human infection model (CHIM) to test the efficacy of vaccines such as *Na*-GST-1/Alhydrogel + CpG, since this will provide convincing evidence of the impact of induced antibodies to the recombinant protein on survival of the worm in a parasitized host.

**References**

1. Diemert DJ, Freire J, Valente V, et al. Safety and immunogenicity of the Na-GST-1 hookworm vaccine in Brazilian and American adults. PLoS Negl Trop Dis **2017**; 11:e0005574.

2. Adegnika AA, de Vries SG, Zinsou FJ, et al. Safety and immunogenicity of co-administered hookworm vaccine candidates Na-GST-1 and Na-APR-1 in Gabonese adults: a randomised, controlled, double-blind, phase 1 dose-escalation trial. Lancet Infect Dis **2021**; 21:275-85.

3. Zinsou JF, Diemert DJ, Dejon-Agobé JC, et al. Safety and immunogenicity of the co-administered Na-APR-1 and Na-GST-1 hookworm vaccines in school-aged children in Gabon: a randomised, controlled, observer-blind, phase 1, dose-escalation trial. Lancet Infect Dis **2024**.

4. Clements AC, Alene KA. Global distribution of human hookworm species and differences in their morbidity effects: a systematic review. The Lancet Microbe **2022**; 3:e72-e9.

5. Zhan B, Liu S, Perally S, et al. Biochemical characterization and vaccine potential of a heme-binding glutathione transferase from the adult hookworm Ancylostoma caninum. Infect Immun **2005**; 73:6903-11.

6. Xiao S, Zhan B, Xue J, et al. The evaluation of recombinant hookworm antigens as vaccines in hamsters (Mesocricetus auratus) challenged with human hookworm, Necator americanus. Exp Parasitol **2008**; 118:32-40.

7. Montano KJ, Cuellar C, Sotillo J. Rodent Models for the Study of Soil-Transmitted Helminths: A Proteomics Approach. Front Cell Infect Microbiol **2021**; 11:639573.
